# Supplementary material for: A single nuclear transcriptomic characterisation of mechanisms responsible for impaired angiogenesis and blood-brain barrier function in Alzheimer’s disease
Source: Nat Commun. 2024 Mar 12;15:2243. doi: 10.1038/s41467-024-46630-z (PMC10933340; doi:10.1038/s41467-024-46630-z)

Supplementary Information

Figure S1. UMAP plots of the integrated snRNAseq dataset (see Figure 1A) by diagnosis (A), sex (B) and brain region (EntC, entorhinal cortex; OC, occipital cortex; OTC, occipital temporal cortex; SSC, somatosensory cortex) showing that the nuclei were well mixed with respect to these parameters after integration. (D) Stacked barplot of the percentage of vascular nuclei from each region. This plot describes a similar relative contribution of each cell type across regions. Abbreviations: AST, astrocytes; EC, endothelial cells; FB, fibroblasts; MGL, microglia; NEU, neurons; NEU, neurons; OLG, oligodendrocytes; PC/SMC, pericytes and smooth muscle cells; LYM, lymphocytes. Source data are provided as a Source data file.

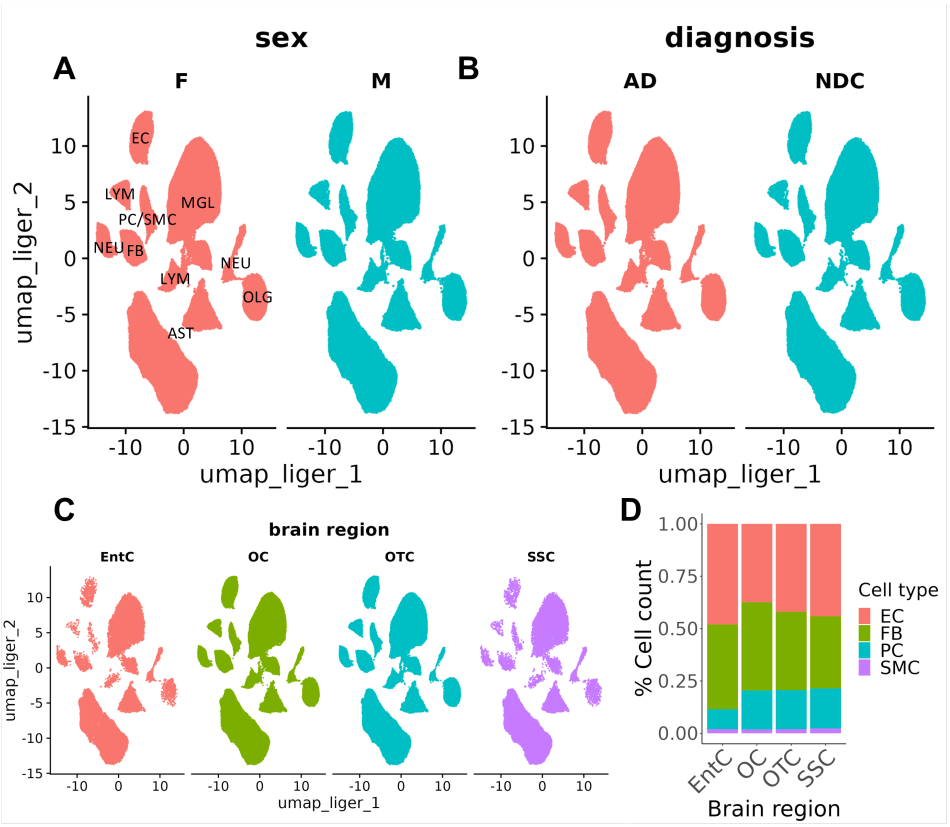

Figure S2. UMAP feature plots of canonical cell marker genes for microglia (*HLA-DRA, C1QB, CD74, CX3CR1, P2RY12*), astrocytes (*AQP4, SCL1A2, GFAP, SLC14A1).* *Cell type labels are provided in Fig. 1A.* Source data are provided as a Source data file.

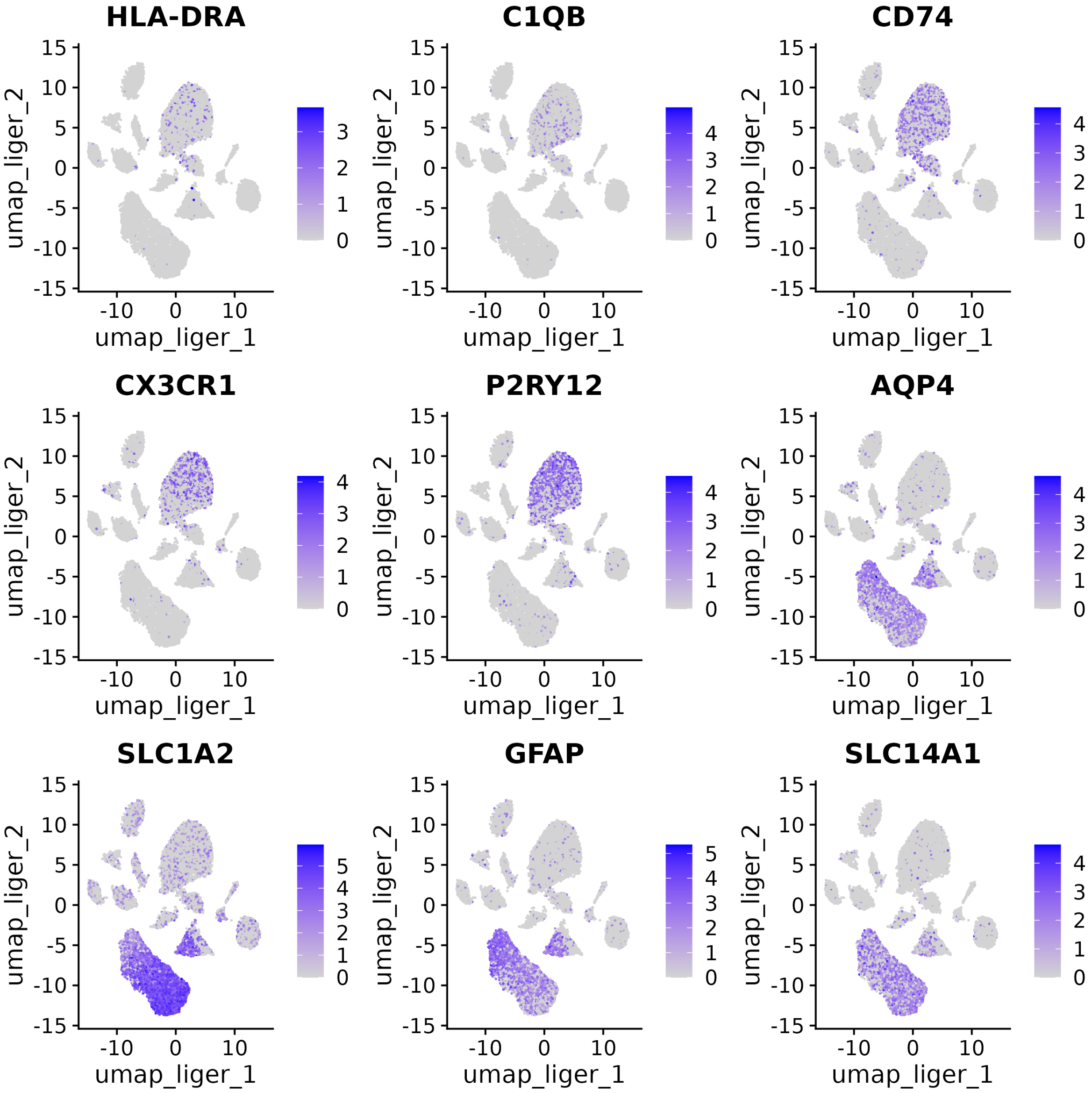

Figure S3. UMAP feature plots of canonical cell marker genes for **oligodendrocytes (*PLP1, MOBP*) and neurons (*GAD1*, *RBFOX3*).** *Cell type labels are provided in Fig. 1A.* Source data are provided as a Source data file.

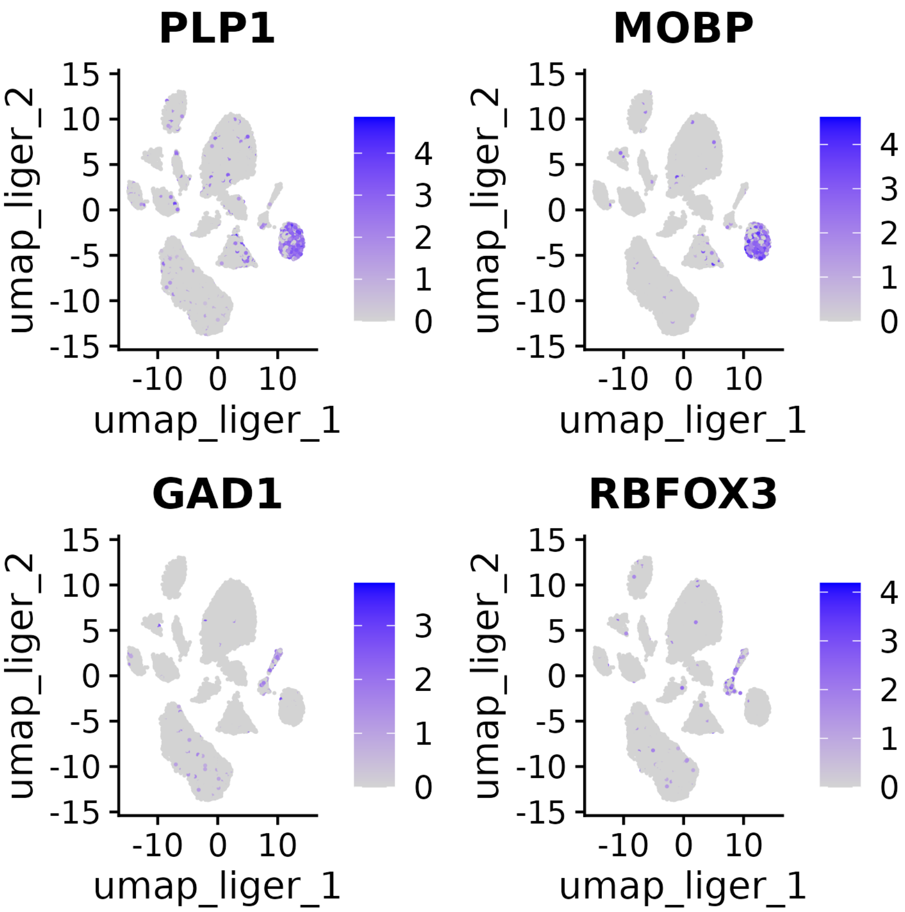

Figure S4. UMAP feature plots of marker genes for EC (A), FB (B) and PC and SMC (C). Note that the UMAP plots are identical to that in Figure 1A but limited to vascular cell clusters. Abbreviations: EC, endothelial cells; FB, fibroblasts; PC/SMC, pericytes and smooth muscle cells. Source data are provided as a Source data file.

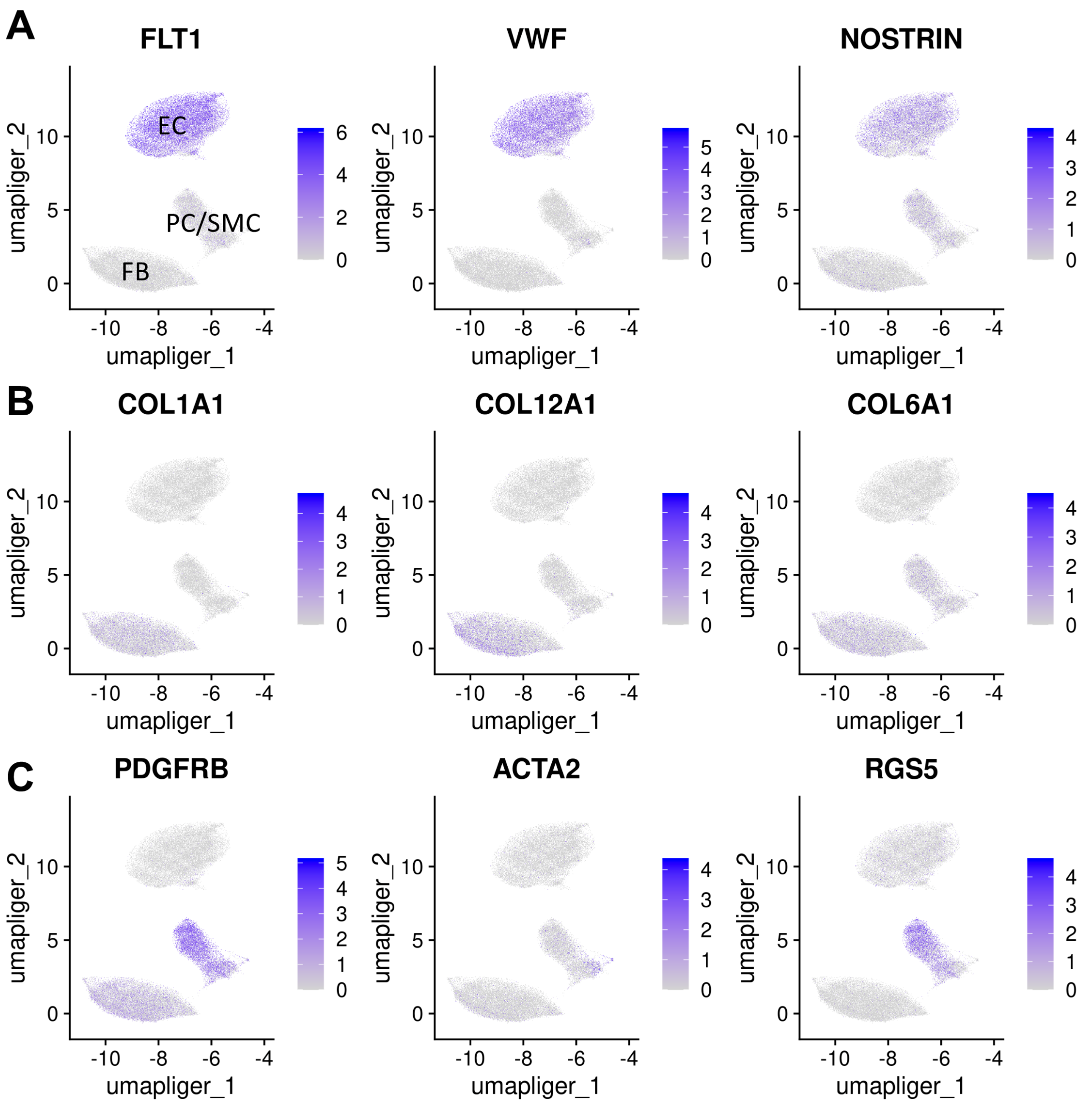

Figure S5. UMAP plot after re-integration and clustering of the EC, FB, PC and SMC nuclei in the integrated dataset (A). EC and FB nuclei are coloured in grey. Four subclusters (PC1, PC2, PC3, PC4 and SMC) correspond to PC and SMC (B). Violin plots of genes previously shown to be specific for PC (*RGS5* and *GRM8)* and SMC *(ACTA2, MYH11). RGS5* and *GRM8* are significantly more highly expressed in PC subclusters compared to SMC (for *RGS5:* PC1 vs SMC: logFC=1.36, padj= 5.23e-96, PC2 vs SMC: logFC=1.41, padj= 7.81e-85, PC3 vs SMC: logFC=1.89, padj= 1.19e-179, PC4 vs SMC: logFC=0.62, padj=1, for *GRM8:* PC1 vs SMC: logFC=2.45, padj= 2.43e-223, PC2 vs SMC: logFC=2.34, padj= 3.23e-183, PC3 vs SMC: logFC=2.92, padj=0, PC4 vs SMC: logFC=1.60, padj= 1.85e-10. *ACTA2* and *MYH11* are significantly more highly expressed in SMC compared to PC (for *ACTA2:* logFC=1.98, padj= 5.86e-147, for *MYH11:* logFC= 3.43, padj=0). Statistical significance was determined using a likelihood ratio test with a mixed-effects model and a zero-inflated negative binomial distribution. The analysis was performed on 10’565 nuclei from 57 independent samples. P values (adjusted) refer to two-sided statistical tests. Source data are provided as a Source data file.

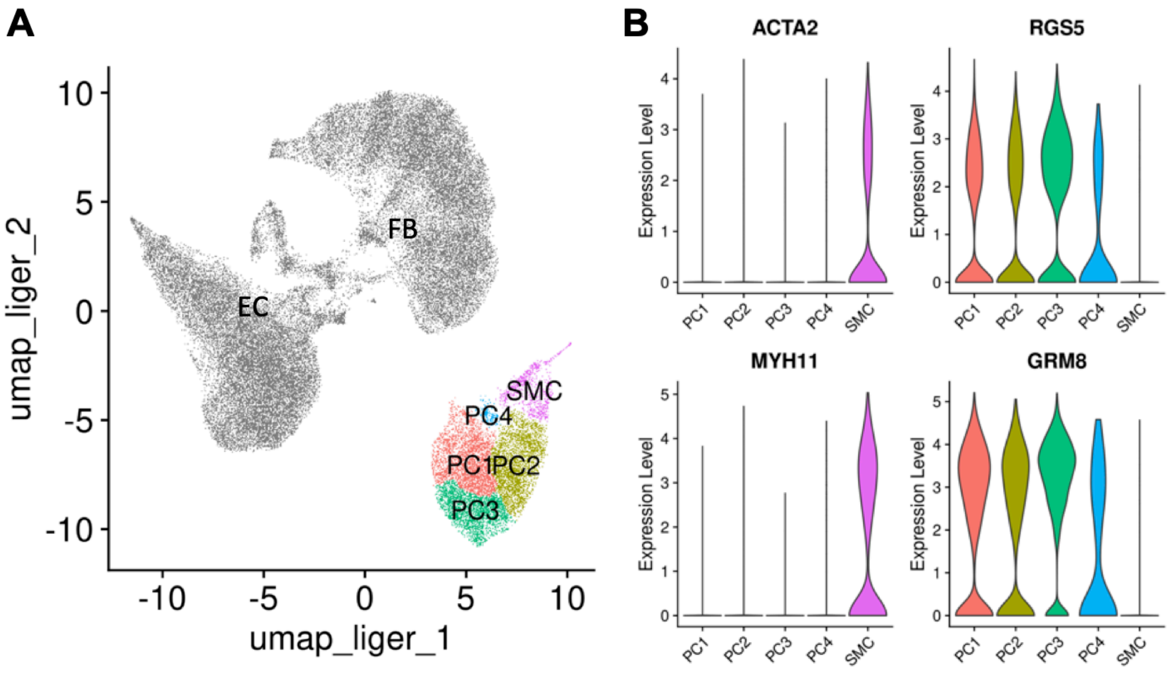

Figure S6 Dot plots of the overlap between cell *subcluster* markers for EC, FB, PC and SMC previously identified in two different snRNAseq studies of the human brain vasculature. (A) EC, PC, SMC and FB subcluster markers from Yang et al[17]. (B) EC, PC and SMC markers from Garcia et al[19]. (C) EC, PC, SMC and FB subcluster markers from Sun et al[26]. The size of the dots correspond to the overlap between the cluster gene sets and the colour of the dot to the adjusted p value (one-sided) of an overrepresentation Fisher's exact test. Source data are provided as a Source data file.

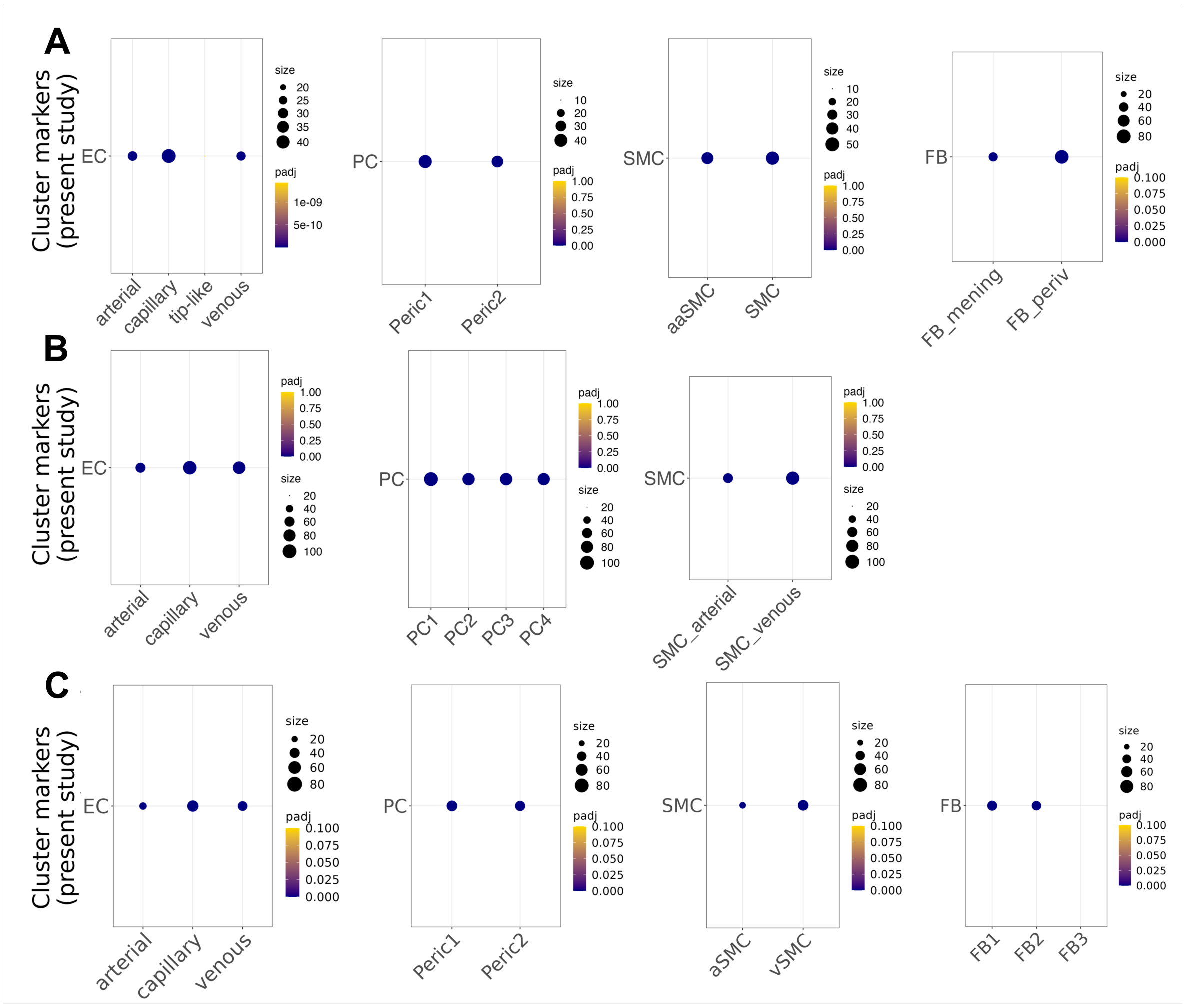

Figure S7. EWCE enrichment of microglia- and astrocyte-specific gene sets*.* Vascular clusters show no enrichment of (A) astrocyte[28] or (B) microglia[27]-specific gene sets, confirming the relative purity of vascular clusters in our dataset. The ordinate provides a measure of the enrichment of each cell type on the abscissa in the gene set considered. It corresponds to the difference, in terms of standard deviations, between the enrichment of each cell type (horizontal axis) and the mean enrichment of the bootstrapped gene sets. P values have been adjusted for multiple comparisons and are one-sided. In (A) the standard deviation of the astrocytic enrichment is 7.19 (padj=0) and in (B) the standard deviation of the microglial enrichment is 11.03 (padj=0). Source data are provided as a Source data file.

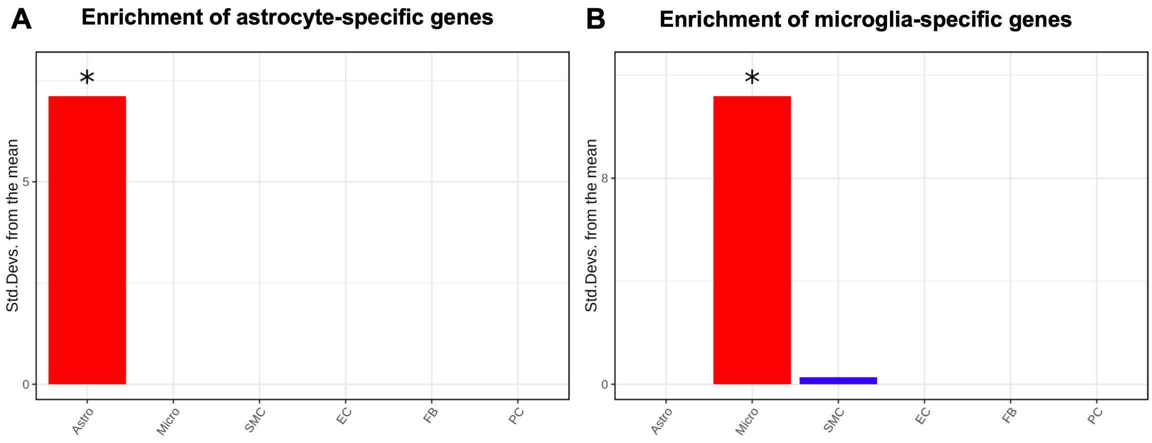

Figure S8 UMAP plot of the snRNAseq dataset that was generated without FACS-enrichment (total brain nuclei dataset). Abbreviations: AST, astrocytes; EN, excitatory neurons; IN, inhibitory neurons; MGL, microglia; OLG, oligodendrocytes; OPC, oligodendrocyte progenitor cells; VASC, vascular cells (including EC, PC, SMC and FB)**.** Source data are provided as a Source data file.

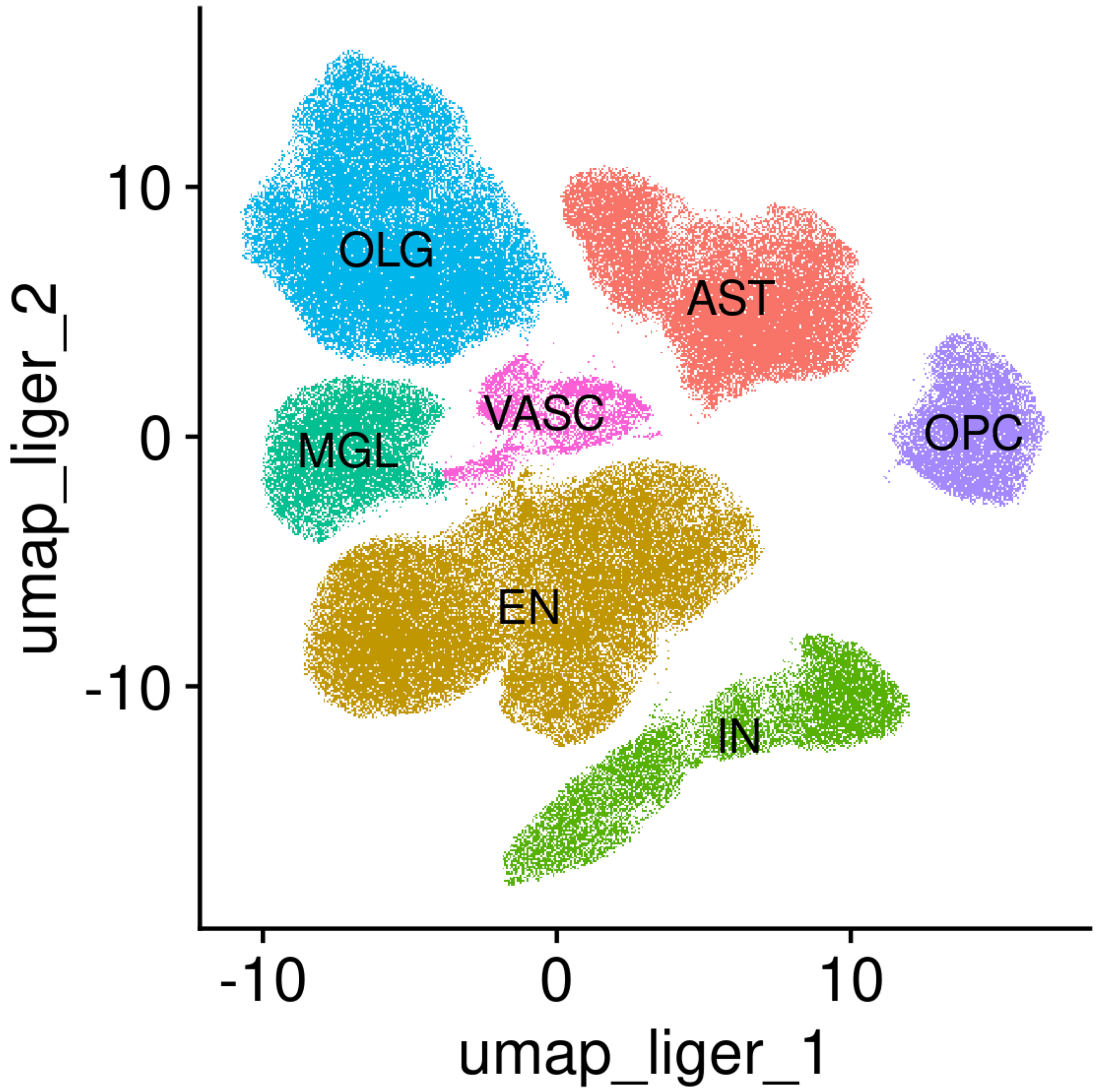

Figure S9 UMAP plots of the snRNAseq dataset that was generated without FACS-enrichment (total brain nuclei dataset) (Figure S9) by diagnosis (A), sex (B) and brain region (EntC, entorhinal cortex; OC, occipital cortex; OTC, occipital temporal cortex; SSC, somatosensory cortex), showing that the nuclei were well mixed with respect to these parameters after integration. Abbreviations: AST, astrocytes; EN, excitatory neurons; IN, inhibitory neurons; MGL, microglia; OLG, oligodendrocytes; OPC, oligodendrocyte progenitor cells; VASC, vascular cells (including EC, PC, SMC and FB)**.** Source data are provided as a Source data file.

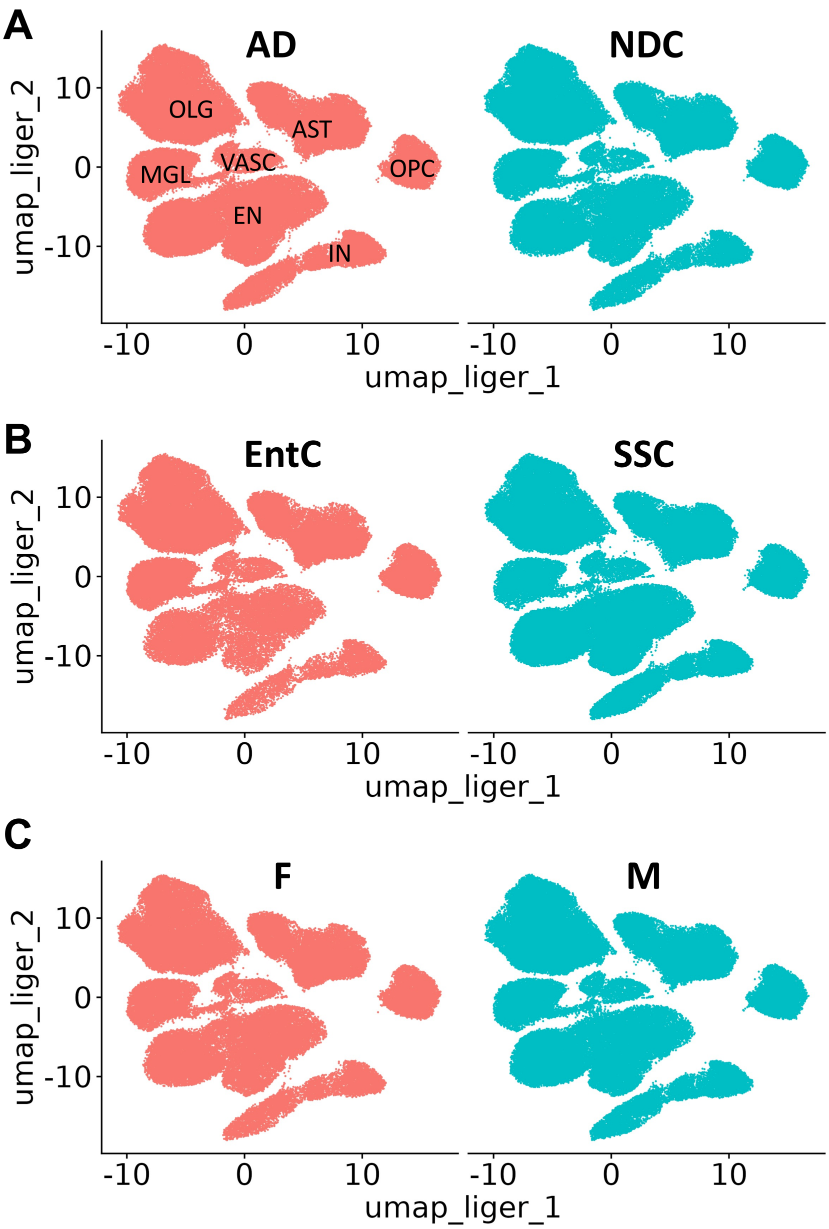

Figure S10 UMAP feature plots of canonical cell marker genes in the total brain nuclei dataset of Figure S9 and S10 for microglia (*CD74*), astrocytes (*GFAP),* oligodendrocytes (*PLP1),* oligodendrocyte precursor cells (*PCDH15*), neurons (*RBFOX3, GAD2, MIAT, MEG3*) and vascular cells (*FLT1, RGS5, ACTA2, COL1A1).* Abbreviations: AST, astrocytes; EN, excitatory neurons; IN, inhibitory neurons; MGL, microglia; OLG, oligodendrocytes; OPC, oligodendrocyte progenitor cells; VASC, vascular cells (including EC, PC, SMC and FB)**.** Source data are provided as a Source data file.

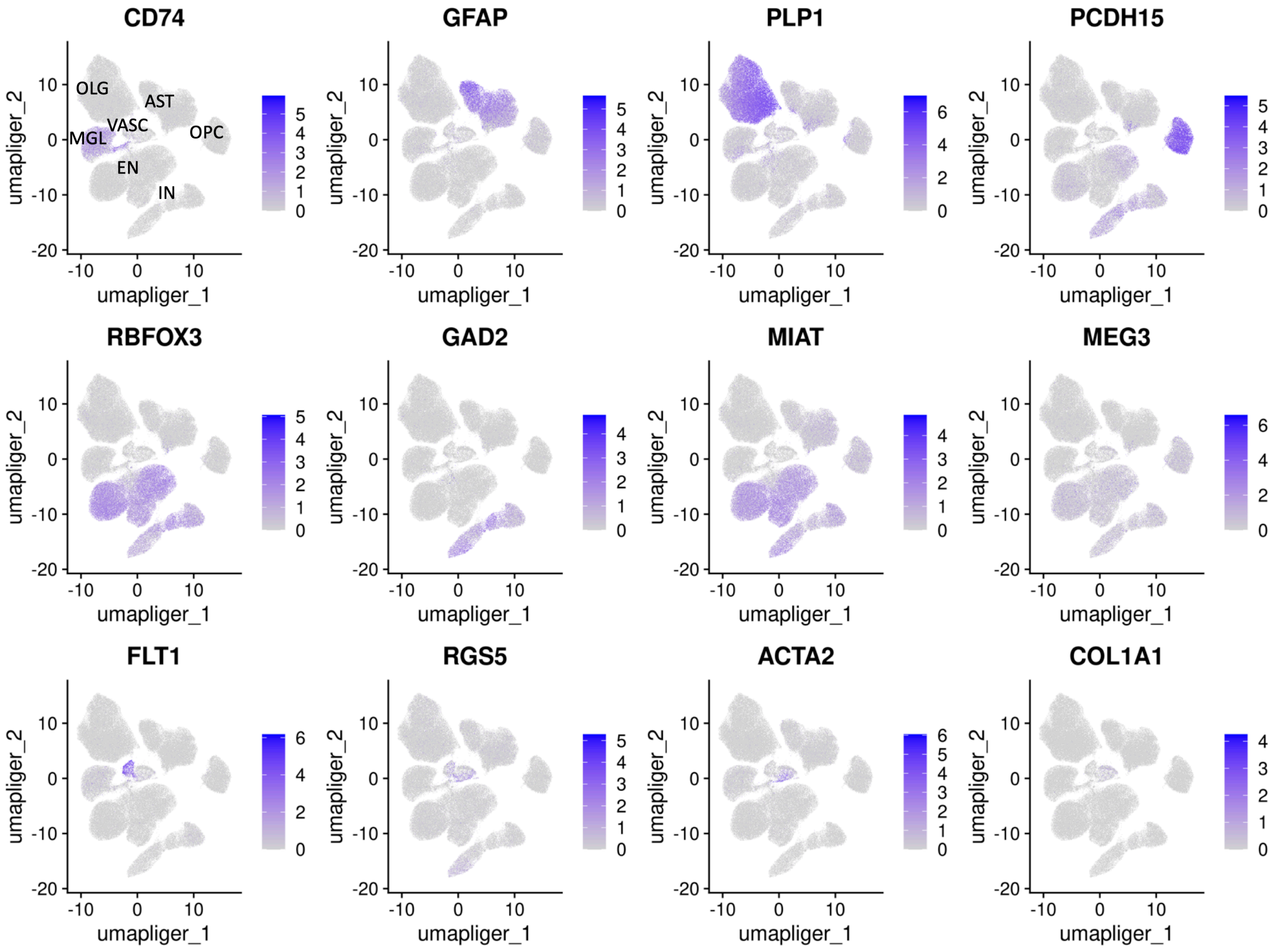

Figure S11 MAGMA.Celltyping enrichment of brain nuclei in genomic loci associated with genetic risk for WMH. (A) The bars correspond to the $\log_{10}$p-value (one-sided) of the enrichement in GWAS signal (i.e. linear regression between cell type specificity of gene expression and the common variant genetic association with the disease using information from all genes. The dark line marks the corrected significance threshold. Only vascular nuclei show enrichment with fully corrected significance across the total brain nuclei dataset. This analysis was performed on 153'128 nuclei from 36 independent samples (B) MAGMA.Celltyping WMH risk gene enrichment of nuclei of the brain vasculature showing no significant enrichment for any of the cell types and that the enrichment for genomic loci associated to WMH is equally distributed among EC, FB and SMC. This analysis was performed on 51'874 nuclei from 57 independent samples. Source data are provided as a Source data file.

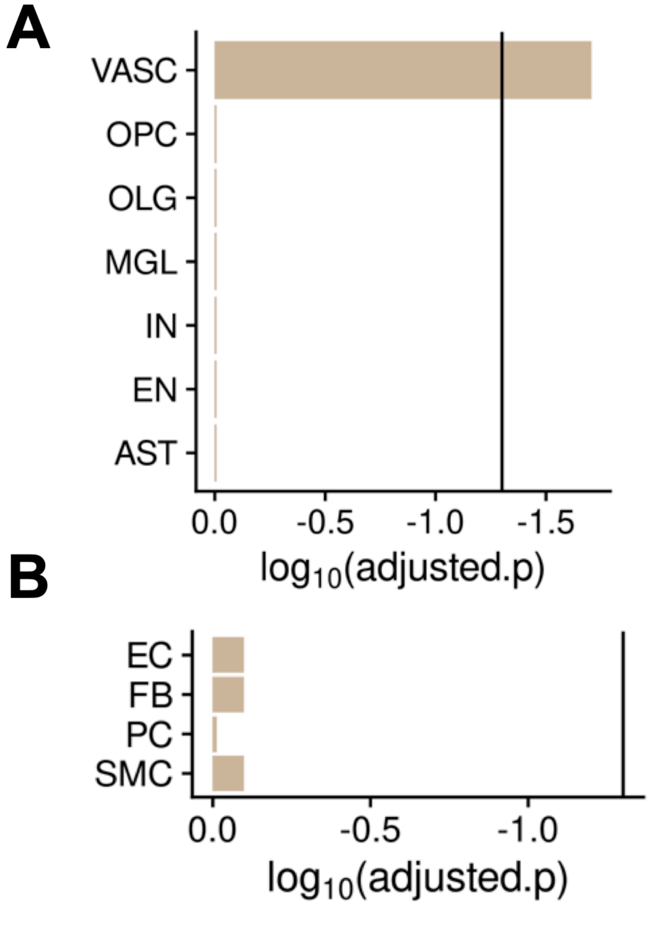

Figure S12 NicheNet intercellular communication analysis of potential regulators of FB DEG (A) Circular heatmap and chord diagram of the results of the NicheNet analysis for FB and (B) PC. The circular plot is divided (based on the innermost track) in ligand genes (black track) and target genes (grey track). The heatmap corresponding to the ligand genes from the 2nd to the 6th innermost tracks represents the average scaled expression of each ligand in each of the "sender" cell types of the vasculature (each cell type is represented on a different track). The two outermost tracks, which correspond to the target genes (grey track), represent the results of the DGE analysis of these genes in the "received" FB (A) or PC (B). The outermost track represented the logFC and the 2nd outermost track represents the $-log_{10}(padj)$ value. The links of the diagram represents the regulatory potential between the ligand and the target genes. Source data are provided as a Source data file.

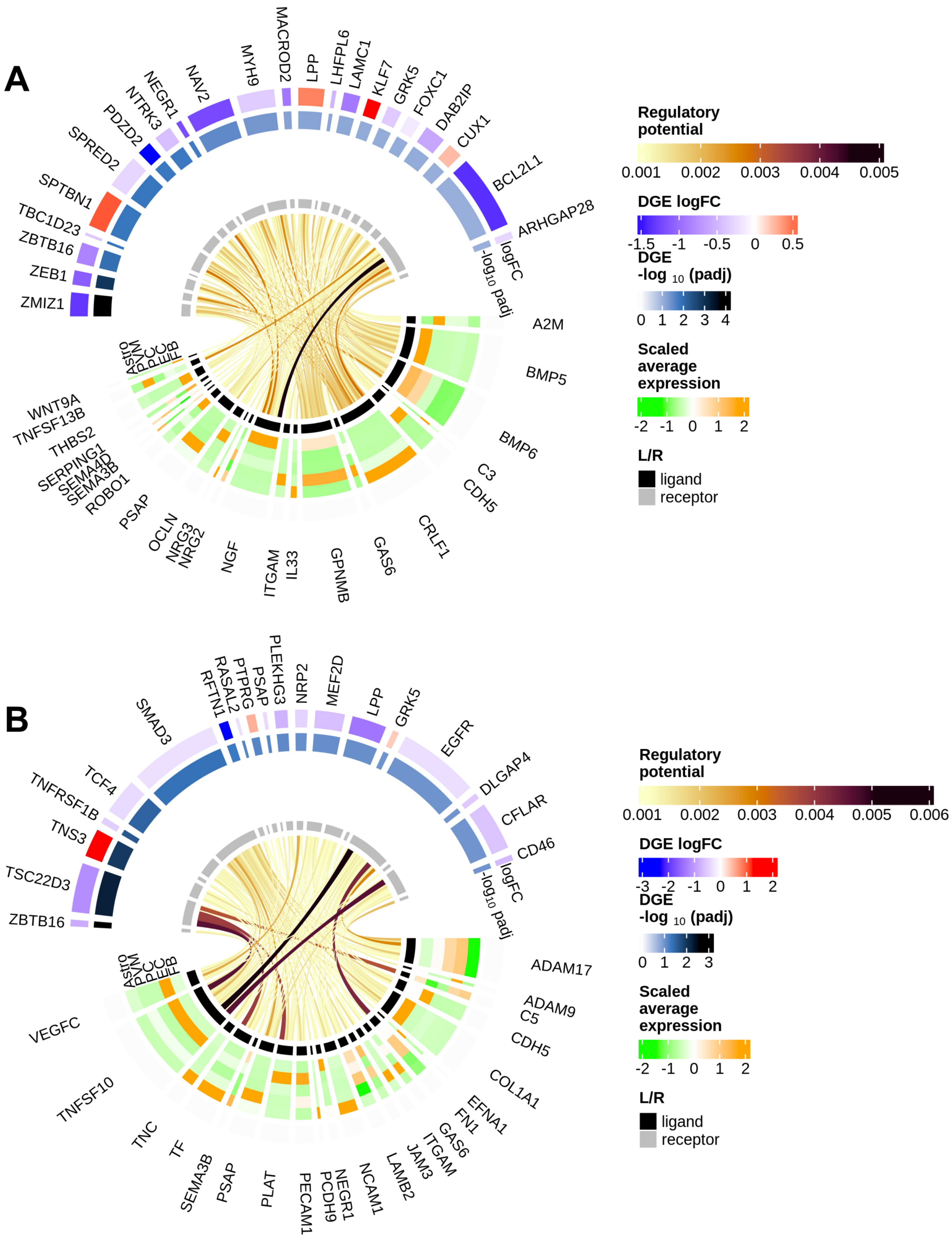
A
ZMIZ1
ZEB1
ZBTB16
TBC1D23
SPTBN1
SPRED2
PDZD2
NTRK3
NEGR1
NAV2
MYH9
MACROD2
LPP
LHFPL6
LAMC1
KLF7
GRK5
FOXC1
DAB2IP
CUX1
BCL2L1
ARHGAP28
logFC
-log10 padj
A2M
BMP5
BMP6
C3
CDH5
CRLF1
GAS6
GPNMB
IL33
ITGAM
NGF
NRG2
NRG3
OCLN
PSAP
ROBO1
SEMA3B
SEMA4D
SERPING1
THBS2
TNFSF13B
WNT9A
Astro
PVM
PC
PEC
FB
Regulatory potential
0.001 0.002 0.003 0.004 0.005
DGE logFC
-1.5 -1 -0.5 0 0.5
DGE -log 10 (padj)
0 1 2 3 4
Scaled average expression
-2 -1 0 1 2
L/R
ligand
receptor
B
ZBTB16
TSC22D3
TNS3
TNFRSF1B
TCF4
SMAD3
RFTN1
RASAL2
PTPRG
PSAP
PLEKHG3
NRP2
MEF2D
LPP
GRK5
EGFR
DLGAP4
CFLAR
CD46
logFC
-log10 padj
ADAM17
ADAM9
C5
CDH5
COL1A1
EFNA1
FN1
GAS6
ITGAM
JAM3
LAMB2
NCAM1
NEGR1
PCDH9
PECAM1
PLAT
PSAP
SEMA3B
TF
TNC
TNFSF10
VEGFC
Astro
PVM
PC
PEC
FB
Regulatory potential
0.001 0.002 0.003 0.004 0.005 0.006
DGE logFC
-3 -2 -1 0 1 2
DGE -log 10 (padj)
0 1 2 3
Scaled average expression
-2 -1 0 1 2
L/R
ligand
receptor

Figure S13 Antibody controls for the IHC experiments. (A) ANG2, (B) (scale bar=50 μm) for (A) ANG2, (B) FGF1, (C) FGFR1, (D) VEGFA, (E) ADAM10. This figure indicates that in the absence of the primary antibody, there is no signal in the tissue sections, supporting the specificity of the antibody binding in the IHC experiment.

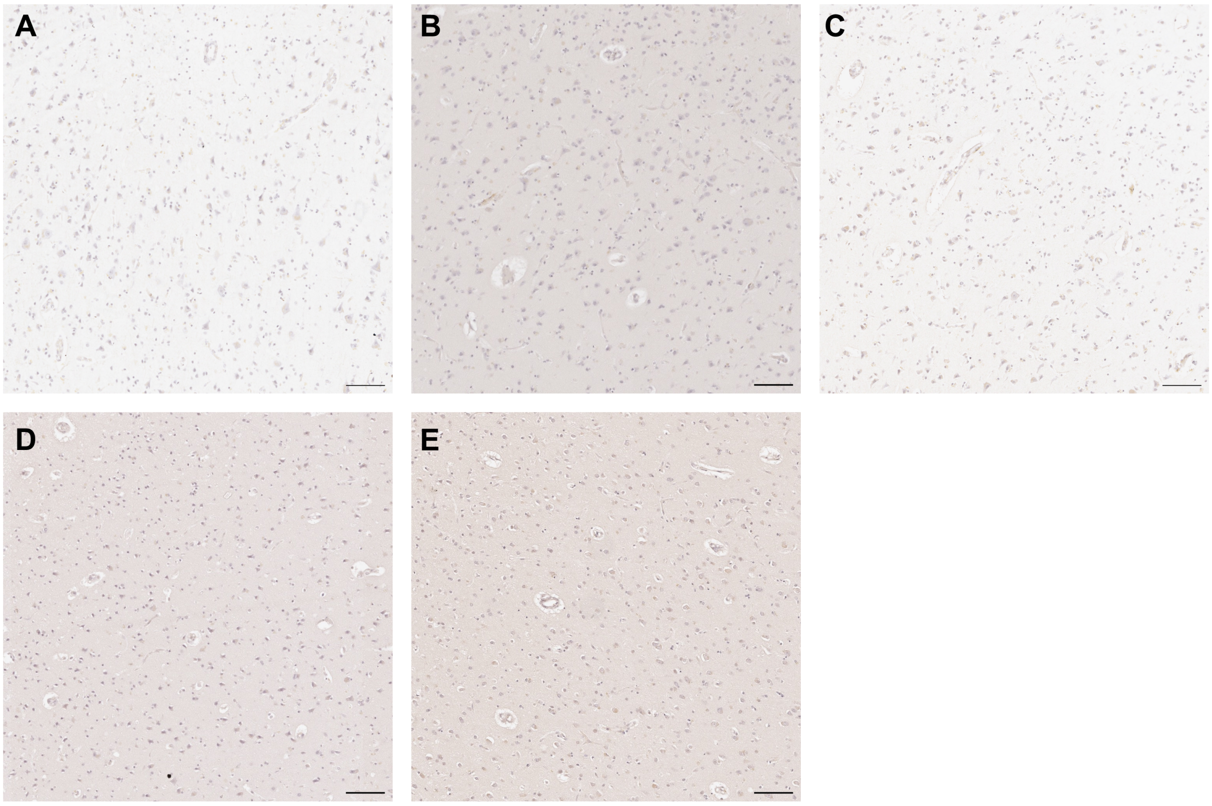

Supplement: Supplementary file 1 — Supplementary Information [file 41467_2024_46630_MOESM1_ESM.pdf]
