# Supplementary material for: A single nuclear transcriptomic characterisation of mechanisms responsible for impaired angiogenesis and blood-brain barrier function in Alzheimer’s disease
Source: Nat Commun. 2024 Mar 12;15:2243. doi: 10.1038/s41467-024-46630-z (PMC10933340; doi:10.1038/s41467-024-46630-z)
Supplement: Supplementary file 3 — Description of Additional Supplementary Files [file 41467_2024_46630_MOESM3_ESM.pdf]

Description of Additional Supplementary Files

File name: Supplementary Data 1

Description: Cluster marker genes

File name: Supplementary Data 2

Description: List of explored GWAS genes

File name: Supplementary Data 3

Description: DEG (AD vs CON) for EC, FB, PC, respective functional enrichment. DEG for the AD vs CON comparison in PVM and astrocytes (employed in the cell-cell communication analysis are also provided

File name: Supplementary Data 4

Description: Co-expression module lists for EC, FB and PC and respective modules tables, indicating characteristics of the modules (e.g., size, hub genes)

File name: Supplementary Data 5

Description: Functional enrichment of co-expression modules that show a significant overrepresentation of DEG for EC, FB and PC

File name: Supplementary Data 6

Description: Results of the fast gene set enrichment analysis for functional pathways upregulated or downregulated in AD in our dataset in comparison to pathways upregulated in a meta-analysis of previously published bulk RNA seq datasets and a snRNAseq dataset. The pval/padj parameters indicate the significance of the enrichment and the ES/NES parameters the effect size of the enrichment.

File name: Supplementary Data 7

Description: List of two-layer neighborhoods of GWAS genes in the EC, FB and PC co-expression networks and functional enrichment of the two-layer neighborhoods.

File name: Supplementary Data 8

Description: Results of the regression analysis between gene expression and Aβ and pTau load in the tissue and the respective functional enrichment. In addition, this file contains the results of the same regression as above, performed separately in APOE-ε4^{+} and APOE-ε4^{-} samples

File name: Supplementary Data 9

Description: Results of the NicheNet analysis for EC, FB and PC. The rank refers to the relative rank of the ligand with respect to the pearson coefficient, that denotes the strength of the association between the ligand and the target set of genes

File name: Supplementary Data 10

Description: List of antibodies and immunostaining methods
