# Supplementary material for: A single nuclear transcriptomic characterisation of mechanisms responsible for impaired angiogenesis and blood-brain barrier function in Alzheimer’s disease
Source: Nat Commun. 2024 Mar 12;15:2243. doi: 10.1038/s41467-024-46630-z (PMC10933340; doi:10.1038/s41467-024-46630-z)
Supplement: Supplementary file 14 — Reporting Summary [file 41467_2024_46630_MOESM14_ESM.pdf]

nature portfolio

Corresponding author(s): Prof Paul Matthews

Last updated by author(s): Feb 16, 2024

Reporting Summary

Nature Portfolio wishes to improve the reproducibility of the work that we publish. This form provides structure for consistency and transparency in reporting. For further information on Nature Portfolio policies, see our Editorial Policies and the Editorial Policy Checklist.

Statistics

For all statistical analyses, confirm that the following items are present in the figure legend, table legend, main text, or Methods section.

n/a	Confirmed	
☐	☒	The exact sample size (*n*) for each experimental group/condition, given as a discrete number and unit of measurement
☐	☒	A statement on whether measurements were taken from distinct samples or whether the same sample was measured repeatedly
☐	☒	The statistical test(s) used AND whether they are one- or two-sided<br>*Only common tests should be described solely by name; describe more complex techniques in the Methods section.*
☐	☒	A description of all covariates tested
☐	☒	A description of any assumptions or corrections, such as tests of normality and adjustment for multiple comparisons
☐	☒	A full description of the statistical parameters including central tendency (e.g. means) or other basic estimates (e.g. regression coefficient) AND variation (e.g. standard deviation) or associated estimates of uncertainty (e.g. confidence intervals)
☐	☒	For null hypothesis testing, the test statistic (e.g. *F*, *t*, *r*) with confidence intervals, effect sizes, degrees of freedom and *P* value noted<br>*Give P values as exact values whenever suitable.*
☒	☐	For Bayesian analysis, information on the choice of priors and Markov chain Monte Carlo settings
☐	☒	For hierarchical and complex designs, identification of the appropriate level for tests and full reporting of outcomes
☐	☒	Estimates of effect sizes (e.g. Cohen's *d*, Pearson's *r*), indicating how they were calculated

Our web collection on statistics for biologists contains articles on many of the points above.

Software and code

Policy information about availability of computer code

Data collection	We used commercial software, as described in Methods to produce the raw count matrices after single nucleus RNA sequencing (using Cell Ranger v3.1). For FACS data, BD FACS DIVA software v9.0 was used.
Data analysis	For data analysis we employed either commercially available or previously described open-source software, as described in Methods: HALO software (Indica Labs, Version 2.3.2989.34) was employed to quantify the immunohistochemistry images. Single nucleus RNA sequencing quality control, dataset integration, dimension reduction and clustering were performed using the Nextflow pipeline nf-core/scflow v0.7.1 (Khozoie et al, Biorxiv) which includes the DropletUtils package(v1.12.1), the DoubletFinder package (v2.0.3) and the riger package (v1.0.0). Further data analysis and visualization was performed using the R package Seurat v3.2.3. Gene overrepresentation analysis was performed using the R package bc3net (v1.0.4) . GWAS summary statistics for AD3 and WMH (a radiological manifestation of small vessel disease)24 were tested for enrichment in brain cell types using the MAGMA.Celltyping (v1.0.1) and MungeSumstats (v1.1.24) R packages. Differential gene expression analysis was performed using the R package MAST (v1.18.0). Gene ontology enrichment and the pathway enrichments analysis were carried out using the R package enrichR (v 3.0) and a hierarchical clustering approach to reduce redundancy in the results as described in Huang et al Genome Biol (see references). Co-expression analysis, module enrichment and statistical comparison of the enrichment between the groups were performed using the MEGENA (v1.3.7), AUCell (v1.6.1) and limma (v3.42.2) R packages, respectively. Expression-weighted celltype enrichmet analysis was performed using the EWCE package (v1.4.0) Cell-cell communication analysis was performed using NicheNet (v1.1.1). The custom code that was employed for this work is available here: https://github.com/stergiostsartsalis/A-single-nuclear-transcriptomic-characterisation-of-mechanisms-responsible-for-impaired-angiogenesis/tree/1

For manuscripts utilizing custom algorithms or software that are central to the research but not yet described in published literature, software must be made available to editors and reviewers. We strongly encourage code deposition in a community repository (e.g. GitHub). See the Nature Portfolio guidelines for submitting code & software for further information.

Data

Policy information about availability of data

All manuscripts must include a data availability statement. This statement should provide the following information, where applicable:

- Accession codes, unique identifiers, or web links for publicly available datasets
- A description of any restrictions on data availability
- For clinical datasets or third party data, please ensure that the statement adheres to our policy

The snRNAseq data are available for download from the Gene Expression Omnibus (GEO) database (https://www.ncbi.nlm.nih.gov/geo/) under accession number GSE160936 (https://www.ncbi.nlm.nih.gov/geo/query/acc.cgi?acc=GSE160936) and GSE252921 (https://www.ncbi.nlm.nih.gov/geo/query/acc.cgi?acc=GSE252921). Previously described data21 was downloaded from the GEO database (GSE148822, https://www.ncbi.nlm.nih.gov/geo/query/acc.cgi?acc=GSE148822). Source data are provided with this paper.

Research involving human participants, their data, or biological material

Policy information about studies with human participants or human data. See also policy information about sex, gender (identity/presentation), and sexual orientation and race, ethnicity and racism.

Reporting on sex and gender	The human tissue that was used in our study comes from brain banks which provide the sex (the biological attribute). Sex was used as a confounder in our analyses, thus our results apply to both sexes (ie refer to common mechanisms between the sexes that account for the differences between Alzheimer's disease and non-demented control samples). The cohort of post mortem samples that we employed was balanced for sex between the diagnoses (Alzheimer's vs non-demented control). Table 1 provides details on the distribution of sexes between the diagnoses. Sex-specific analyses were out of the scope of this study.
Reporting on race, ethnicity, or other socially relevant groupings	There were no socially constructed or socially relevant categorization variables used in our study.
Population characteristics	Samples were obtained from post mortem tissue provided by people who volunteered for sharing their tissues in brain banks in the UK. Characteristics: For the transcriptomics study: Non-diseased controls: 41 samples, age at death: 80.61 +/- 6.3, Alzheimer's disease: 36 samples, age at death: 78.47 +/- 9.3. For the ELISA experiments: Non-diseases controls: 4 samples, age at death: 86.75 +/- 8.3, Alzheimer's diseae: 6 samples, age at death: 78.66 +/- 9.6
Recruitment	Brain samples were selectedfrom brain banks, based on the presence of AD-compatible neuropathology (AD group) without significant co-morbid disease pathology or, alternatively, the absence of neuropathological or clinical evidence of neurological disease (NDC group).
Ethics oversight	The experiments at Imperial College London were performed in accordance with National Research Ethics Committee approvals through the UK Brain Banks providing them and approvals of the UK Human Tissue Authoity with governance oversight from by Imperial College London. Ethics oversight of the experiments performed by Gerrits et al (Acta Neuropathol 2021) are described in the published manuscript

Note that full information on the approval of the study protocol must also be provided in the manuscript.

Field-specific reporting

Please select the one below that is the best fit for your research. If you are not sure, read the appropriate sections before making your selection.

☒ Life sciences ☐ Behavioural & social sciences ☐ Ecological, evolutionary & environmental sciences

For a reference copy of the document with all sections, see nature.com/documents/nr-reporting-summary-flat.pdf

Life sciences study design

All studies must disclose on these points even when the disclosure is negative.

Sample size	This study integrates data from two different cohorts, one produced by our group and a second published earlier this year and referenced in the text. Together, this generated the largest dataset(n=57) to date based on Fluorescence-activated sorting (FACS) enrichment of nuclei isolated before snRNAseq r to achieve a better representation of the less abundant brain cell types of interest including brain vasculature-associated cells.
Data exclusions	Three samples were excluded because 1) in one of them (sample from the entorhinal cortex of the A096/14 individual) the nf-core/scflow software detected serious quality issues with single nucleus rna sequencing that would bias the results. Indeed, it had a particularly low number of detected distinct features and it also showed an aberrant gene expression profile. Indeed, the nf-core/scflow pipeline performs PCA plots of the pseudo-bulk global gene expression profiles per sample. This sample was the only one that did not cluster with any other of the samples of the study and showed a vary distinct global gene expression profile. 2) Two samples from the same individual (entorhinal and somatosensory cortex from individual A319/11) were excluded because ulterior genotyping analyses demonstrated that this individual was carrying an AD-associated TREM2 gene variant (R62H).

Replication	For the transcriptomics analysis, we assessed the overlap between our results and the results of previously published datasets of vascular snRNAseq in Alzheimer's disease (as described in the results). We also allow to download the integrated analysis pipeline in Docker and describe parameter choices to allow other to repeat out analysis on our dataset or on future data that becomes available. We further tested for consistency of main observations by employing a range of analytical strategies: 1) differential gene expression analysis, 2) gene co-expression analysis, 3) regression of gene expression with brain pathology. In addition, we employed statistical approaches that reduce type I error (ie a mixed-effects model for the differential gene expression and gene regression analysis and the duplicate correlation function in the differential co-expression module enrichment analysis).<br>Finally, for the IHC experiments, each experiments was performed on 24 biological replicates (12 per diagnosis group). All these are described in Methods and in the results.
Randomization	The design does not employ randomisation; it employs a case-matched control comparisons in which samples of the two clinically and pathologically groups and age- and sex-matched. Covariates such as the brain region and sex were included as confounders in the statistical models of the differential gene expression and regression analysis and the co-expression module differential enrichment analysis.
Blinding	Research team members generating the data from the biological samples were blinded to the case or control origin of individual datasets. Case-control definitions were linked to these data only at the stage of bioinformatic processing by a separate group of bioinformaticists .

Reporting for specific materials, systems and methods

We require information from authors about some types of materials, experimental systems and methods used in many studies. Here, indicate whether each material, system or method listed is relevant to your study. If you are not sure if a list item applies to your research, read the appropriate section before selecting a response.

Materials & experimental systems

n/a	Involved in the study
☐	☒ Antibodies
☒	☐ Eukaryotic cell lines
☒	☐ Palaeontology and archaeology
☒	☐ Animals and other organisms
☒	☐ Clinical data
☒	☐ Dual use research of concern
☒	☐ Plants

Methods

n/a	Involved in the study
☒	☐ ChIP-seq
☐	☒ Flow cytometry
☒	☐ MRI-based neuroimaging

Antibodies

Antibodies used

For each antibody used in IHC experiments, the following information is provided: Antigen, Antibody, Dilution, Antigen Retrieval method, IHC Staining Kit

Ab, 4G8 (BioLegend-800702), 1:15,000, Citrate Buffer in Steamer, Supersensitive Kit

pTau AT8 ((Invitrogen -MN1020) , 1:1,600 , Citrate Buffer in Steamer, Supersensitive Kit

VEGFA, Abcam (ab52917), 1:100 EDTA in Steamer, ImmPRESS DAB Peroxidase Vector Labs

FGF2 , Abcam (ab246354), 1:2000, EDTA in Steamer, ImmPRESS DAB Peroxidase Vector Labs

FGFR1, Novus (M19B2) 1:300, EDTA in Steamer, ImmPRESS DAB Peroxidase Vector Labs

Ang-2, Biotechne (MAB0983), 1:300, EDTA in Steamer, ImmPRESS DAB Peroxidase Vector Labs

ADAM10 , Protein tech (66620-1-Ig) 1:1000, EDTA in Steamer , ImmPRESS DAB Peroxidase Vector Labs

For ELISA experiments, the following antibodies were used:

AB42: R&D systems, https://www.rndsystems.com/products/human-amyloid-beta-aa1-42-quantikine-elisa-kit_dab142, 1:625-2'500

MAG antibody (Abcam), 1:1'000

PLP1 antibody: SEA417Hu; Cloud-Clone Corp., USA/China

fibrinogen: fibrinogen EH3057; FineTest, China

CD31 DY806-05; R&D systems, USA

CD105, KE00199; Proteintech, USA

Validation

For the IHC experiments.

Aβ: 4G8: https://www.biolegend.com/nl-be/products/anti-beta-amyloid-17-24-antibody-10999?GroupID=BLG15648, validated in Alzheimer's disease human brain tissue. It as been very extensively publishes

pTau: AT8: extensively validated in the literature, in human brain tissue: https://absoluteantibody.com/product/anti-phospho-tau-at8/

VEGFA, human kidney tissue IHC was used as positive control according ot the manufacturerhttps://www.abcam.com/products/primary-antibodies/vegfa-antibody-ep1176y-c-terminal-ab52917.html

FGF2: Human prostate and cerebrum tissue (https://www.abcam.com/products/primary-antibodies/fgf2-antibody-ep1735-bsa-and-azide-free-ab246354.html)

FGFR1, https://www.novusbio.com/products/fgfr1-antibody-m19b2_nb600-1287af488, binding confirmed in canine IHC experiments

Ang-2, reactivity was confirmed in human IOHC experiments https://www.bio-techne.com/p/antibodies/human-angiopoietin-2-antibody-180102_mab0983

ADAM10 , https://www.ptglab.com/products/ADAM10-Antibody-66620-1-Ig.htm, IHC reactivity was confirmed in human prostate cancer tissue

For all IHC antibodies, to confirm antibody specificity, the exact same staining protocol was applied without the addition of the primary antibody (described in results)

For ELISA experiments:

AB42, R&D systems, https://www.rndsystems.com/products/human-amyloid-beta-aa1-42-quantikine-elisa-kit_dab142, validated in multiple publications in human tissue lysate, cell culture supernates and cerebrospinal fluid

MAG antibody (Abcam), validation has been extensively described in the literature by the co-authors (JS Minrs, S Love): Miners, S., Moulding, H., de Silva, R. & Love, S. Reduced vascular endothelial growth factor and capillary density in the occipital cortex in dementia with Lewy bodies. Brain Pathol 24, 334-343 (2014). https://doi.org:10.1111/bpa.12130
116 Barker, R., Wellington, D., Esiri, M. M. & Love, S. Assessing white matter ischemic damage in dementia patients by measurement of myelin proteins. J Cereb Blood Flow Metab 33, 1050-1057 (2013). https://doi.org:10.1038/jcbfm.2013.46
117 Barker, R. et al. Pathophysiology of white matter perfusion in Alzheimer's disease and vascular dementia. Brain 137, 1524-1532 (2014). https://doi.org:10.1093/brain/awu040
118 Miners, J. S., Palmer, J. C. & Love, S. Pathophysiology of Hypoperfusion of the Precuneus in Early Alzheimer's Disease. Brain Pathol 26, 533-541 (2016). https://doi.org:10.1111/bpa.12331

PLP1: The manufacturer's website (https://www.cloud-clone.com/products/SEA417Hu.html) cites:"This assay has high sensitivity and excellent specificity for detection of Proteolipid Protein 1, Myelin (PLP1).
No significant cross-reactivity or interference between Proteolipid Protein 1, Myelin (PLP1) and analogues was observed." Multiple citations are also provided in the website,

fibrinogen: https://www.fn-test.com/product/eh3057/: multiple publications where this kit was used are cited

CD31; https://www.rndsystems.com/products/human-cd31-pecam-1-duoset-elisa_dy806-05: Reactivity was confirmed using human CD31, whereas specificity was tested using mouse and porcine CD31 according to the manufacturer's website

CD105: https://www.ptglab.com/products/Human-Endoglin-CD105-ELISA-Kit-KE00199.htm#product-information reactivity was confirmed using human CD105, according to the mnufacturer's website

Plants

Seed stocks: NA

Novel plant genotypes: NA

Authentication: NA

Flow Cytometry

Plots

Confirm that:

- [x] The axis labels state the marker and fluorochrome used (e.g. CD4-FITC).
- [x] The axis scales are clearly visible. Include numbers along axes only for bottom left plot of group (a 'group' is an analysis of identical markers).
- [x] All plots are contour plots with outliers or pseudocolor plots.
- [x] A numerical value for number of cells or percentage (with statistics) is provided.

Methodology

Sample preparation	Described in detail in Methods ("Nuclei isolation and enrichment for lower abundancy cell populations") as initially performed in Smith et al (Acta Neuropathol, In Press and as a pre-print doi.org/10.1101/2021.07.19.452932)
Instrument	BD Aria II
Software	BD FACSDiva v9.0
Cell population abundance	described in detail in Smith et al (Acta Neuropathol, In Press and as a pre-print doi.org/10.1101/2021.07.19.452932)
Gating strategy	described in detail in Smith et al (Acta Neuropathol, In Press and as a pre-print doi.org/10.1101/2021.07.19.452932)

- [ ] Tick this box to confirm that a figure exemplifying the gating strategy is provided in the Supplementary Information.
